# Telemonitoring System Oriented towards High-Risk Pregnant Women

**DOI:** 10.3390/healthcare10122484

**Published:** 2022-12-08

**Authors:** Mirna Arlene Robles Cuevas, Ismael López Martínez, Eduardo López Domínguez, Yesenia Hernández Velázquez, Saúl Domínguez Isidro, Luis Manuel Flores Frías, Saúl Eduardo Pomares Hernández, María Auxilio Medina Nieto, Jorge de la Calleja

**Affiliations:** 1 Laboratorio Nacional de Informática Avanzada, Veracruz 91100, Mexico; 2 Centro de Investigación y de Estudios Avanzados del Instituto Politécnico Nacional, Ciudad de Mexico 07360, Mexico; 3 Faculty of Statistics and Informatics, Universidad Veracruzana, Xalapa, Veracruz 91020, Mexico; 4 Hospital Angeles Xalapa, Veracruz 91193, Mexico; 5 Instituto Nacional de Astrofísica, Óptica y Electrónica (INAOE), Puebla 72840, Mexico; 6 Postgraduate Department, Universidad Politécnica de Puebla (UPPuebla), Puebla 72640, Mexico

**Keywords:** high-risk pregnancy, mobile web applications, telemonitoring system

## Abstract

A high-risk pregnancy is one in which pathological problems or abnormal conditions are latent during pregnancy and childbirth, increasing dangers to the mother’s or the infant’s health. Based on international standards and studies, most of the harms and risks to both the mother and the infant can be detected, treated, and prevented through proper pregnancy monitoring, as well as through appropriate and timely diagnosis. In this paper, we present the analysis, design, development, and usability assessment of a telemonitoring system focused on the remote monitoring and control of pregnancy in women suffering from hypertension, diabetes, or high-risk pregnancy. Our system is composed of two mobile web applications. One of these is designed for the medical area, allowing remote monitoring of the patient’s pregnancy, and the other one is directed towards the patient, who enters the alarm symptom data, hypertension data, diabetes data, and clinical analyses, allowing the detection of a risk situation on time. Furthermore, we performed a usability assessment of our system based on a laboratory study with seven doctors and seven patients to evaluate the users’ satisfaction. Our telemonitoring system shows a satisfactory/favorable opinion from the users’ perspectives based on the obtained results.

## 1. Introduction

According to a study presented in 2019 by the World Health Organization (WHO), approximately 295,000 women died from complications during pregnancy and childbirth in 2017 [1]. A high-risk pregnancy (HRP) is one in which there are pathological problems or abnormal conditions latent during pregnancy and childbirth, increasing the dangers to the mother’s and the infant’s health. Among the main factors that generate high-risk pregnancies are the health problems existing in women before their pregnancy, such as diabetes and hypertension [2,3,4,5]. High blood sugar levels in the mother can cause birth defects during the first weeks of pregnancy, often even before the woman knows she is pregnant [3]. On the other hand, in the case of pregnant women with uncontrolled high blood pressure, there is a risk of causing damage to the mother’s kidneys and increasing the risk of pre-eclampsia or having an infant with low body weight [4]. The WHO indicates that these deaths could have been avoided, with quality care before, during, and after pregnancy [6]. Likewise, the Official Mexican Standard NOM-007-SSA2-2016 establishes that risks can be prevented or decreased through the proper monitoring of pregnancy, as well as more timely diagnoses [7]. It also establishes that such attention must be carried out during pregnancy, delivery, and puerperium. An alternative to carrying out this monitoring and control is through telemonitoring. Telemonitoring is defined as the set of support systems and medical services that perform remote monitoring of a patient’s situation and their vital parameters, allowing the aid of provision and health care to patients in their usual environment [8]. Some works [9,10,11,12,13,14,15,16,17,18,19,20,21,22,23,24,25] have proposed systems that monitor hypertensive, diabetic, and/or high-risk pregnant women. However, these systems lack important services, such as managing a patient’s medical history, identifying a possible risk situation, notifying the responsible physician in real time, receiving medical recommendations, and monitoring the infant, among others.

In this work, we present the analysis, design, development, and usability assessment of a telemonitoring system focused on the remote monitoring and treatment of pregnant women suffering from hypertension, diabetes, and other risk factors such as severe anemia, morbid obesity, mild pre-eclampsia, gestational diabetes, cardiopathy II (slight limitation of physical activity), previous uterine surgery, twin or multiple pregnancies, endocrinological diseases, second and/or third trimester hemorrhage, hydramnios or oligohydramnios, unfavorable obstetric history (e.g., two or more miscarriages, one or more premature births), and maternal infections. The analysis and design of the proposed system consider the requirements outlined in the manual of the electronic clinical record of the Ministry of Health (Mexico) [26], as well as some of the design considerations presented in [27,28,29]. The core of our system (alerts of risk situations and medical recommendations) was based on the Official Mexican Standard NOM-007-SSA2-2016, interviews with medical specialists in gynecology, and a group of mothers who were at risk during their pregnancy. Therefore, our telemonitoring system consists of two applications: a mobile web application that offers various services to the patient, and a mobile web application that provides services oriented toward the specialist doctor. Analysis, design, and development models presented in this work can serve as a reference point for system developers looking to improve or develop telemonitoring systems [30]. Finally, we performed a usability assessment of our system based on a laboratory study with seven doctors and seven patients to evaluate the users’ satisfaction [31]. Our telemonitoring system shows a satisfactory/favorable opinion from the perspectives of the users based on the obtained results.

## 2. Materials and Methods 

The complete development of the telemonitoring system was divided into two stages, which were developed based on the ICONIX methodology [28] as a formal development methodology.

### 2.1. Analysis and Design Models

In this methodology, analysis and design models are applied. These include use case diagrams and a data model. In the following subsections, each diagram is detailed.

#### 2.1.1. Use Case Diagrams

**Figure 1:**
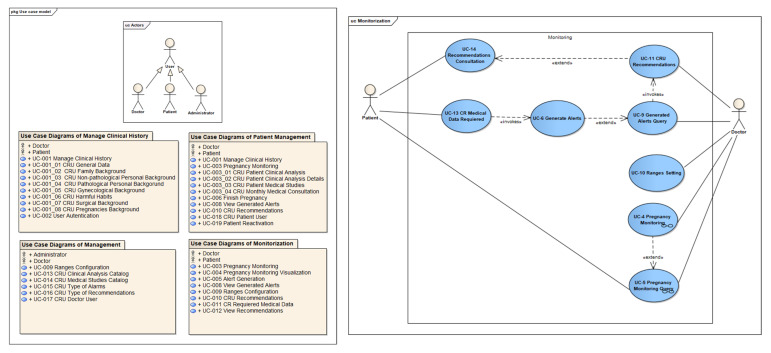
Package diagram of use cases (**left**) and use case diagram for monitoring (**right**).

Our use case diagrams were developed based on the guidelines proposed in the ICONIX methodology [28]. These guidelines define a series of specific steps to be carried out to adequately identify actors, scenarios, and use cases of the system. Figure 1 shows the analysis package diagram of the proposed use cases for our telemonitoring system. In Figure 1, the doctor, patient, and administrator actors can be observed: (1) the doctor actor is a gynecology specialist with an intermediate level of digital skills; (2) the patient actor is a woman with a pregnancy greater than four weeks of gestation with a basic level of digital skills; and (3) the administrator actor can be a medical specialist with an intermediate or advanced level of digital skills. Likewise, subsystems that involve UCs for the management of the patient’s medical history, administration of the information about the patient pregnancy, telemonitoring of patients’ pregnancy, and finally, the UCs for the system management were introduced. On the other hand, Figure 1 shows the use case diagram that corresponds to the monitoring subsystem, which includes UCs regarding the control and telemonitoring of risk situations that could occur during the patient’s pregnancy. This diagram is composed of eight UCs, and the involved actors are the doctor and the patient. It starts by capturing recommended medical data, and from these, the red, yellow, or green alerts are generated.

Once the data are captured, alerts are created, according to a symptom configuration that permits classifying alerts (see Appendix A). Based on this identification and classification of risk situation alerts presented in Appendix A, if a hypertensive pregnant patient enters the following symptom data from its application, temperature of 38.5 degrees Celsius, blood pressure (systolic/diastolic) of 160/110 mm Hg, and heart rate of 105 bpm, our system identifies that the patient is in a possible risk situation and automatically generates a red alert to the doctor with this information. Once the alerts are produced, the medical part generates a recommendation to the patient, as a response to the generated alarm or just to provide medical support to the patient. Finally, the patient consults the recommendations made by the medical part to act on it.

#### 2.1.2. Data Model

**Figure 2:**
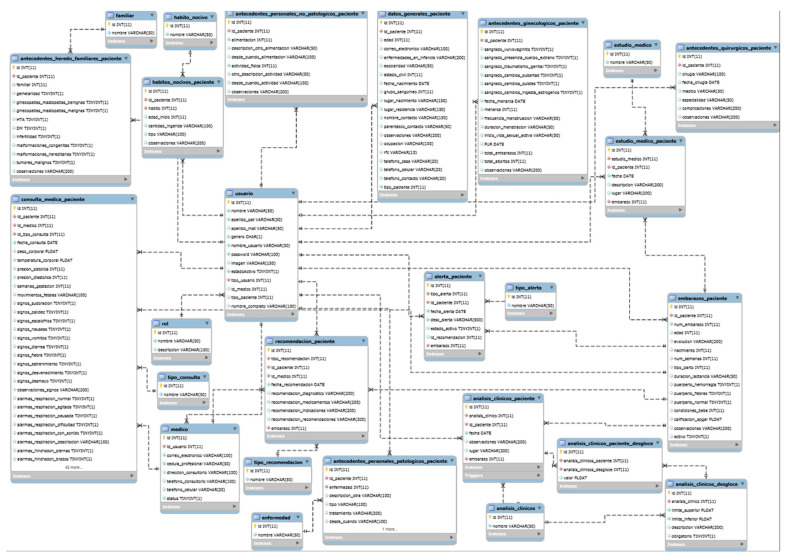
Data model of the telemonitoring system.

Figure 2 shows the data model of our telemonitoring system aimed at pregnant women, which stores the information sent by the doctor and patient applications, respectively. The main tables of our data model are described below:

- Patient_Alerts: This table stores the alarms generated by general symptoms, hypertension symptom data, and/or diabetes symptom data of the patient. In this table, the alert_type attribute takes the following values: 1 (Red Alert), 2 (Yellow Alert), 3 (Green Alert).
- Patient_Clinical_Analysis: This table manages the patient clinical analyzes.
- Family_Background: This table stores the family background of the patient.
- Gynecological_Background: This table stores the gynecological background of the patient.
- Non-pathological_Personal_Background: This table stores the patient’s non-pathological personal background.
- Pathological_Personal_Background: This table stores the patient’s pathological personal background.
- Surgical_Background: This table manages the patient’s surgical background.
- Harmful_Habits_Background: This table controls the history of the patient’s harmful habits.
- Pregnancies_Background: This table stores the patient’s pregnancy history.
- Medical_Study: Table that administers the medical studies of the patient.
- Recommendation_Patient: This table stores the recommendations generated by the doctor to the patient.
- Consult_Type: This table manages the catalog of consult types.
- Users: This table manages the system users.
- Patient_General_Data: This table collects general patient information.
- Medical_Consultation: This table contains the history of the patient’s consults and is related to the Users table, in addition to saving the identification of the doctor who generated the consult relating to the medical table by means of the medical_id attribute.

The data model was designed to store the continuous data generated in both applications, to facilitate maintenance and to preserve the integrity of the data generated. Figure 2 shows a relational model. In our case, to consult the general data of the patients, a query is made in the Users table, which has an attribute that indicates whether the user is a patient and through the identification is related to the Patient_General_Data table. On the other hand, if the patient’s clinical record needs to be consulted, the Users table is related to the tables Patient_Clinical_Analysis, Family_Background, Gynecological_History, Non-pathological personal history, Pathology_Personal_History, Surgical_Background, Harmful_Habits_Background, Pregnancies_Background, and Medical_Study. These tables are updated during medical consultations and medical analyses. On the other hand, the records that the doctor makes during pregnancy monitoring are stored in the Medical_Consultation tables where, based on the Consult_Type catalog, it is possible for the doctor to indicate the reason for the consultation, as well as to generate recommendations that are stored in the Recommendation_Patient table in response to the alarms generated. Finally, when the patient records alarm, diabetes, and hypertension symptoms, a record is generated in the Medical_Consultation table with the query type “alert” which allows the system to generate a record in the Patient_Alerts table.

## 3. Results: Telemonitoring System

Our telemonitoring system consists of two mobile web applications. One of these is designed for the medical area, allowing remote monitoring of the patient’s pregnancy, and the other one is directed towards the patient. The main services implemented in the proposed system are described below.

### 3.1. Doctor Application Services

The physician user is responsible for the management and telemonitoring of the patient. Therefore, the main functions of this type of user are listed below:

- Patient reactivation
- Patient management
- Create new patient
- Register patient
- Edit patient
- Finished pregnancy
- Clinical history management
- Medical Consultation
- Clinical Analysis
- Medical Studies
- Alarms
- Recommendations
- Alarms Visualization
- Catalogs

The most significant services are described below.

#### 3.1.1. Alarm Visualization

**Figure 3:**
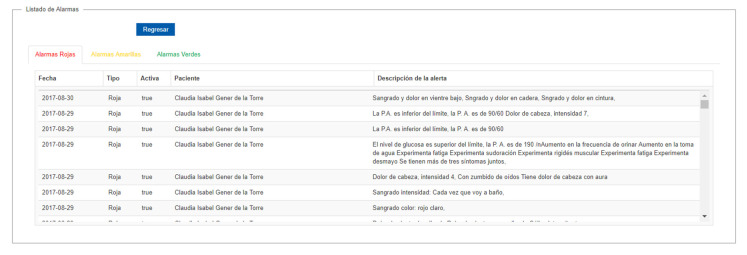

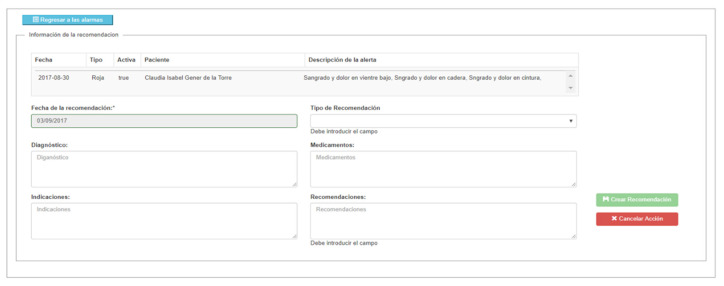
List of medical alarms generated by patients and medical recommendation edition.

Figure 3 shows the alarms that are generated by the patients, which are ordered according to the relevance level, starting with the red alarms, followed by the yellow ones, and finally the green ones. The red alarms are the most important because they can detect a situation of imminent risk for the patient or the infant and must be attended immediately by the doctor. On the other hand, the yellow alarms are of medium importance, since they can indicate the beginning of a risk situation and must be attended by the doctor at the time, he considers necessary. Finally, green alarms are informational only and tell the doctor if the patient entered information. Figure 3 presents information from a test patient user.

#### 3.1.2. Recommendations

A recommendation can be created in two ways, in response to an alarm or simply as an additional recommendation for the patient. Every time a recommendation is generated, a text message and an email are sent to the patient, see Figure 3.

### 3.2. Patient Application Services

The services provided by the application to the patient are as follows:

- Record alarm symptoms.
- Record hypertension symptom data.
- Record diabetes symptom data.
- Consult clinical history.
- Record clinical analysis.
- Record medical studies.
- View medical consultation.
- View medical recommendations.

The first three services are guided questionnaires with the aim of obtaining medical information that may lead timely to the detection of risk situations. The medical information of the patients in our system is classified into three categories based on the type of patient. Each category of medical information is directed towards a type of patient according to the health standard [7]. However, the patient can register information in all the services of our system, since her state of health could eventually present symptoms that she did not initially have, such as diabetes or hypertension. Therefore, in our system, the recording of information is determined by the instructions of the specialist doctor based on the patient’s state of health. It is noted that each category was reviewed and validated by a group of medical specialists to detect risk situations using the information recorded by the patient. These categories are described below:

- Symptoms of hypertension: this category is aimed at pregnant patients with arterial hypertension problems.
- Symptoms of diabetes: this category is aimed at diabetic pregnant patients.
- Alarm symptoms: this category is aimed at patients with high-risk pregnancies caused by different conditions to diabetes and hypertension, such as severe anemia, morbid obesity, cardiopathy II (slight limitation of physical activity), previous uterine surgery, twin or multiple pregnancies, endocrinological diseases, second and/or third trimester hemorrhage, hydramnios or oligohydramnios, unfavorable obstetric history (e.g., two or more miscarriages, one or more premature births), and maternal infections (hepatitis B or C virus, toxoplasmosis, pyelonephritis, rubella, syphilis, HIV infection, streptococcus B).

The most important services are described below.

#### 3.2.1. Record Diabetes Symptom Data

**Figure 4:**
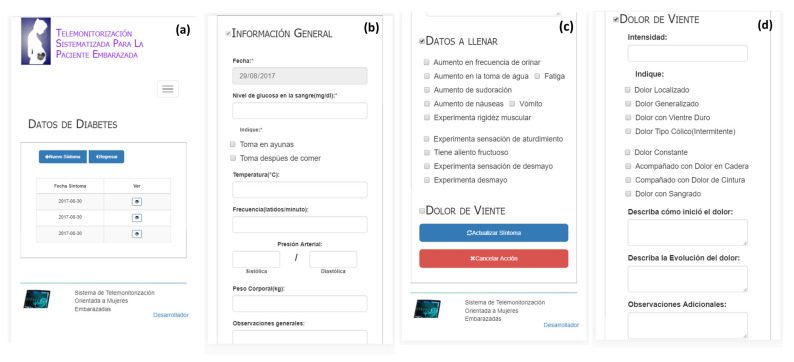
Diabetes symptom data (from left to right): (**a**) list, (**b**) general information, (**c**) data to fill out, and (**d**) belly pain data.

The diabetes symptom data are the medical data required mainly for patients with this health problem, and the information contained generates alarms. Figure 4 shows the screen system of the list and the diabetes symptom data sections: general information (Figure 4b), data to fill out (Figure 4c), and belly pain data (Figure 4d). 

To register a new diabetes report, the patient should fill out the general information section (see Figure 4b), which includes the blood glucose level, indications about drink habits (on an empty stomach or after eating), temperature, heart rate, blood pressure (systolic and diastolic), weight, and general observations. Likewise, Figure 4c displays a list of information that should be indicated by the patient: increase in urination frequency, increase in water intake, fatigue, increase in sweating, increase in sickness, vomiting, muscular stiffness, stunned sensation, fruitful breath, feeling faint, and fainting. Finally, the user interface, with regards to the belly pain section (see Figure 4d), collects the pain intensity as well as a different class of pain information, such as localized, generalized, coinciding with a hard belly, intermittent colic, constant, accompanied with hip pain, accompanied with waist pain, and pain with bleeding, and ending with a description of how the pain starts, the pain evolution, and additional observations.

#### 3.2.2. Record Hypertension Symptom Data

**Figure 5:**
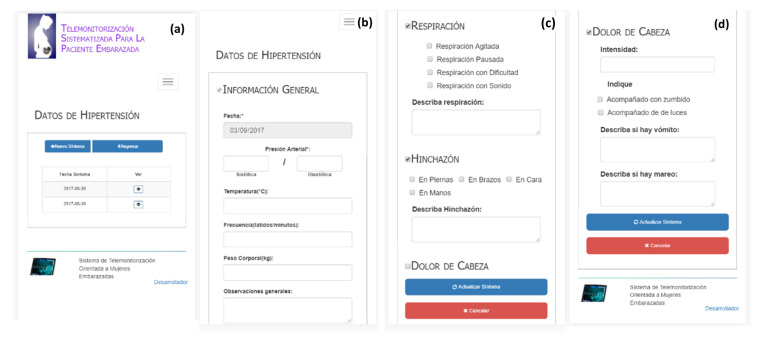
Hypertension symptom data (from left to right): (**a**) list, (**b**) general information, (**c**) breathing and swelling data, and (**d**) headache data.

Figure 5 shows the screen system of the list and the hypertension symptom data sections: general information (Figure 5b), breathing and swelling data (Figure 5c), and headache data (Figure 5d). To register a new hypertension report, the patient should fill out the general information section (see Figure 5b), which includes blood pressure (systolic and diastolic), temperature, heart rate, weight, and general observations. Likewise, the system allows the collection of breathing information (see Figure 5c). Figure 5c also shows the section of Swelling data registration (legs, arms, face, and hands). Finally, in the section of Headache data registration shown in Figure 5d, the patient should describe the intensity, indicate whether it is accompanied with buzzing or with lights, and detail whether vomit and dizziness are occurring.

#### 3.2.3. Record Alarm Symptoms

**Figure 6:**
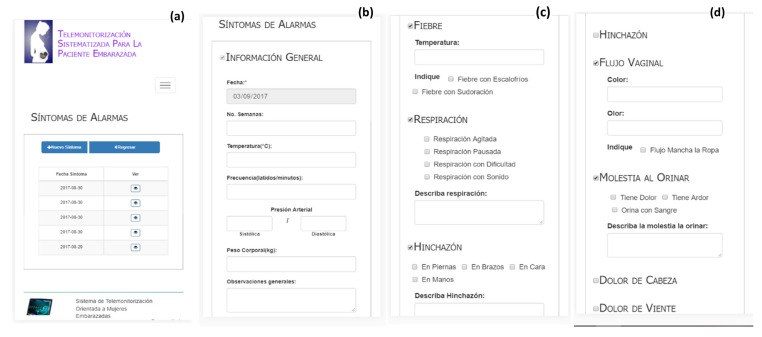
Alarms symptoms (from left to right): (**a**) list, (**b**) general information, (**c**) fever, breathing, and swelling data, and (**d**) vaginal fluid data and discomfort during urination.

**Figure 7:**
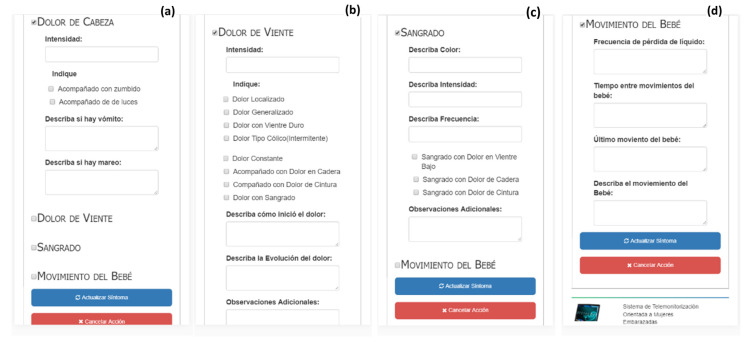
Alarms symptoms (from left to right): (**a**) headache data, (**b**) belly pain data, (**c**) bleeding data, and (**d**) infant movement data.

Alarms symptoms are the medical data required mainly for patients with high-risk pregnancies caused by different conditions to diabetes and hypertension, such as severe anemia, morbid obesity, cardiopathy II (slight limitation of physical activity), previous uterine surgery, twin or multiple pregnancies, endocrinological diseases, second and/or third trimester hemorrhage, hydramnios or oligohydramnios, unfavorable obstetric history (e.g., two or more miscarriages, one or more premature births), and maternal infections. Each time an alarm is generated, a text message and an email are sent by the system to the doctor. Figure 6 and Figure 7 show the list and sections of the alarm symptoms of the proposed system.

To register a new alarm symptom the patient should fill out (type) the general information section (see Figure 6b), which includes temperature, heart rate, blood pressure (these data are obtained with a thermometer and baumanometer), weight, and general observations. Figure 6c presents the user interface to collect data regarding fever (fever with shivers or sweating). Likewise, the system allows collecting data on breathing (restless or slow breathing and breathing with difficulty or with sound). Finally, swelling data are collected to indicate the extremities where it occurs (legs, arms, face, and hands). Figure 6d shows the sections to collect data about vaginal fluid and discomfort during urination. 

Regarding the section of headache data registration shown in Figure 7a, the patient should indicate the intensity, whether it is accompanied with buzzing or with lights, and describe whether vomiting and dizziness are present, respectively. The user interface, with regards to belly pain data (see Figure 7b), collects the pain intensity and diverse class of pain. Finally, the patient can describe how the pain starts, the pain evolution, and additional observations. The bleeding data section shown in Figure 7c allows patients to describe the color, intensity, and frequency of bleeding, as well as indicate whether the bleeding is accompanied with pain in the lower belly, with hip paint, or with waist pain, and ending with any additional observations. Finally, the user interface to register data concerning infant movements (see Figure 7d) allows patients to describe four situations: frequency loss of amniotic fluid, the time between movements, the last movement, and the movement description. In this service, patients will fill in the information with the frequency that the doctor suggests or as the symptoms show up.

#### 3.2.4. Record Clinical Analysis

**Figure 8:**
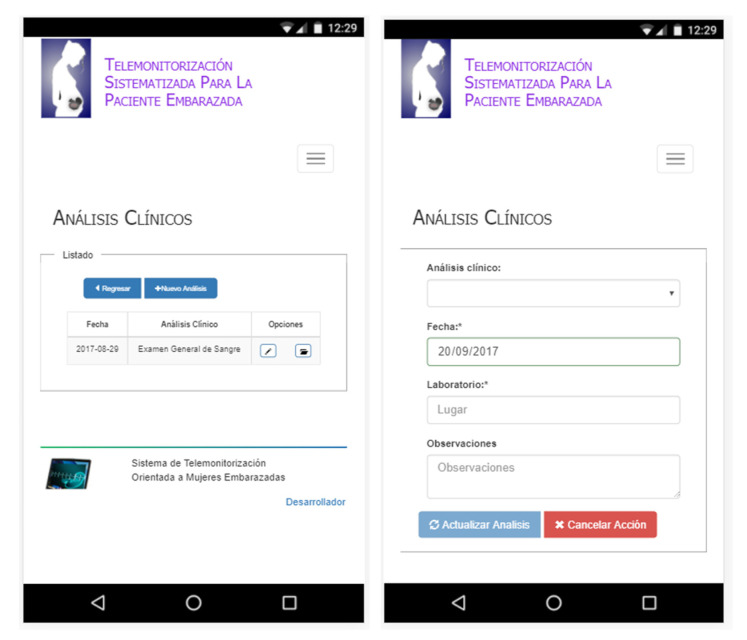
Clinical analyses (from **left** to **right**): list and general record.

In this service, the patient records the results of clinical analyses that are performed based on the indications of the medical specialist (see Figure 8). In our case, the proposed system provides a configuration service that manages an extensive catalogue of various clinical analyses and medical studies, which were defined based on the risk factors addressed in this work.

#### 3.2.5. View Medical Recommendations

**Figure 9:**
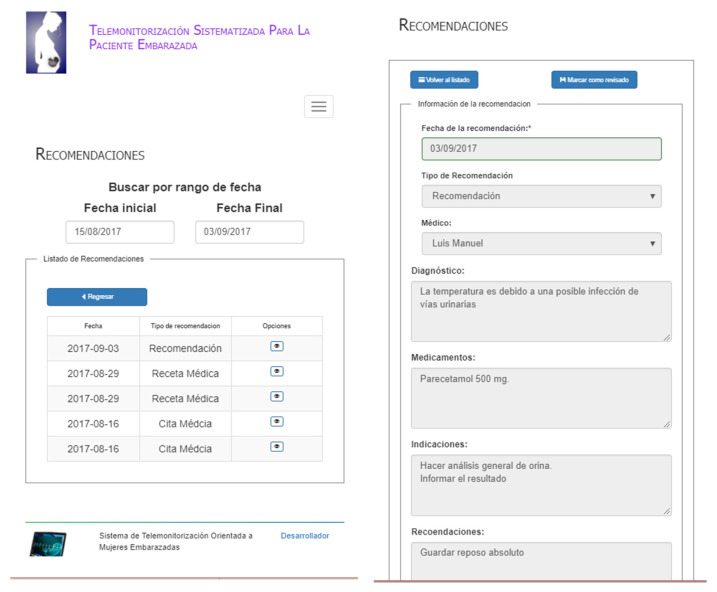
List and access to recommendations.

Figure 9 shows the medical recommendations addressed to the patient during the monitoring process. The patient will always view the recommendations of the current date. However, there is the option to select medical recommendations in a date range. It is worth mentioning that the patient does not perceive whether a medical alarm is generated when she enters the medical data because it could generate a state of anxiety in the patient, for this reason, she only receives the recommendation (see Figure 9).

In all the system services proposed in this work, contextual help is provided to the user to guide them on the information they must enter and/or report. The system also performs internal validations of numerical, floating, and alphanumeric data, and ranges of acceptable values to guarantee the correct entry of information by the patient or doctor. Additionally, the system allows the doctor to verify and/or modify, during the patient’s physical visit, any information entered incorrectly by the patient, for example, the results of medical studies.

On the other hand, the services developed in both web applications were reviewed and validated by patients (hypertensive, diabetic, and/or high-risk pregnant women) and doctors (specialists in gynecology and obstetrics) of the Hospital Ángeles Xalapa, who also contributed to the analysis and the list of requirements initially requested. In the analysis phase, we considered specialized documents and guides taken from the World Health Organization (WHO) and Official Mexican Standard NOM-007-SSA2-2016 [1,7]; hence, our system is aligned with international and national standards.

### 3.3. Usability Assessment

We conducted a usability assessment of the proposed system in this work by applying a laboratory study [31] with fourteen users: seven doctors (specialists in gynecology and obstetrics) with an intermediate level of digital skills and seven patients (hypertensive, diabetic, and/or high-risk pregnant women) with a basic level of digital skills. Informed consent was obtained from all subjects involved in the laboratory study. The participants were asked to individually perform different activities associated with the services provided by their applications. Finally, following the guidelines of the usability standard ISO/IEC 25010:2011 [32], the users’ satisfaction was assessed by applying the questionnaire QUIS 7.0 [33,34]. This questionnaire consists of a set of questions grouped into aspects such as (a) general reaction to the software; (b) system user interface (Screens); (c) terminology and system information; (d) learning; (e) system capabilities, and (f) technical manuals and online help. In this instrument, the Likert scale selected for each question consists of ten conceptual levels of satisfaction, where 0 points are the lowest rating, and 9 points represent the highest rating. Once user answers are compiled, the average of each category is estimated, as well as the total average. Based on [35], the acceptability ranges are as follows:

*0 ≤ satisfaction ≤ 3: unsatisfactory*.
*4 ≤ satisfaction ≤ 6: acceptable*.
*7 ≤ satisfaction ≤ 9: satisfactory*.

The results obtained based on the responses of the QUIS 7.0 satisfaction questionnaire of the patients and doctors are presented below.

#### 3.3.1. Doctor User Satisfaction Results

**Figure 10:**
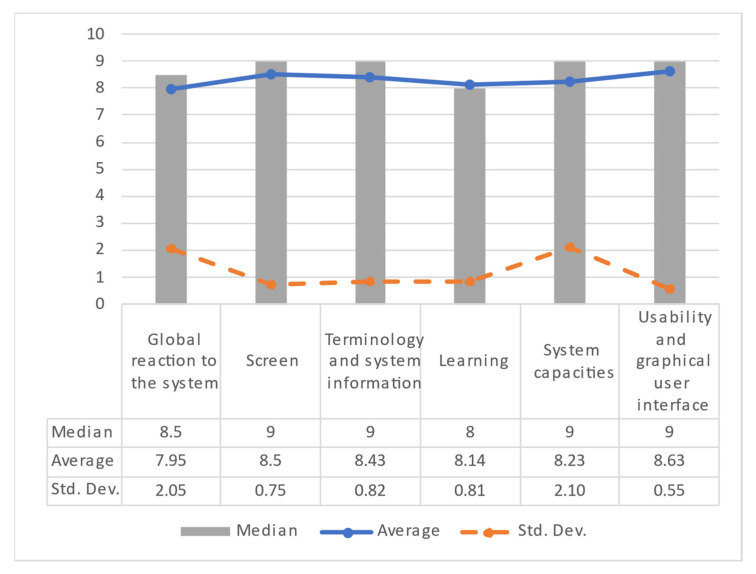
Doctor user satisfaction results by category.

Based on these results, the average, median, and standard deviation (Std. Dev.) were computed to measure the level of satisfaction of all medical users. Regarding the results summarized in Figure 10, the closeness between the mean and median values for each usability dimension indicates a positive tendency in terms of satisfaction. In addition, the standard deviations have values less than 1, showing coincident results from four usability aspects on the part of the medical staff.

Finally, concerning the global reaction to the system, one of the users rated the system as acceptable, which is reflected in the data variance. Despite this, the results show a satisfactory level of system usage from the doctors’ point of view.

#### 3.3.2. Patient User Satisfaction Results

**Figure 11:**
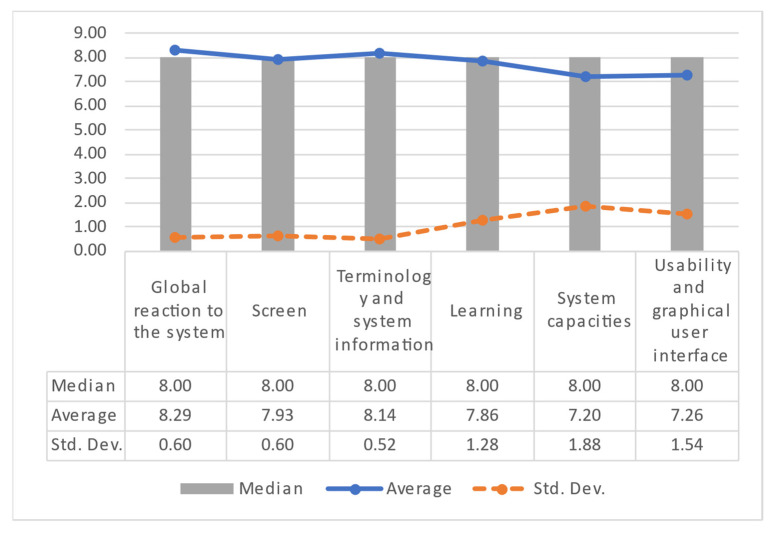
Patient user satisfaction results by category.

Concerning the patient users, the average, median, and standard deviation (Std. Dev.) of each application category are summarized in Figure 11. The median of the patients’ scores provided to the system is 8 in all dimensions. In this regard, there exists a marginal difference (less than one) regarding the average values, indicating a positive satisfaction trend. However, there are three categories in which the variance of the data is higher than 1. This is because, in these dimensions, the patients scored the system with acceptable values due to the application’s learning curve. Nevertheless, the system has a satisfactory score from the patients’ point of view.

Finally, and according to the analysis carried out in [36], the number of users who participated in our study allowed us to identify probably around 90% of the usability problems in both applications of our system. The main usability issues identified in this laboratory study were confusing information architecture and navigation sequence in some services provided to doctors and patients. The usability issues identified in our study were fixed.

## 4. Discussion 

The proposed system rises with the need to optimize the monitoring of high-risk pregnancies considering various aspects involved in the control of this type of pregnancy. Both in the literature [13,14,15,16,17,18,19,20,21,22,23,24,25] and the market applications [9,10,11,12], there are several systems addressed towards self-care and monitoring of pregnancies, which offer biosignal control services and data related to risk conditions during pregnancy, helping clinicians and patients to take timely actions. In this regard, we analyzed a set of telemonitoring systems proposed in the literature and founded in the application market, based on the following aspects (see Table 1):

- Patient application (PA): application that can be used by patients to monitor their pregnancy.
- Doctor application (DA): application that can be used by physicians to monitor and control the pregnancy of their patients.
- Clinical history (CH): provides the patient’s medical history.
- Medical recommendations (MR): the physician can make medical recommendations and the patient receiving them, it involves a bidirectional communication between the clinician and patient.
- Alarms generation (AG): alarms or alerts are generated indicating if there is any possible risk during the pregnancy period.
- Maternal monitoring (MM): the monitoring of the patient’s health during pregnancy is carried out.
- Fetal monitoring (FM): the monitoring of the child’s development during pregnancy is carried out.
- Data view (DV): information such as medical consultations or medical records can be visualized at any moment.

These aspects were selected based on the guidelines established in the Official Mexican Standard NOM-007-SSA2-2016 [7] and feedback provided by specialist doctors and patients.

Overall, the purpose of all the cited approaches is to obtain patient information and deliver it to the physician for possible monitoring, control, and remote treatment. However, none of these systems achieve the aim completely, since they lack basic services, such as clinical history management. Hence, a complete patient history is not supported by most existing systems, and thus potential risk situations are not reported to the specialist. Furthermore, other important services, such as medical recommendations and fetal monitoring do not always take place in the market applications. The most complete analyzed system [19] requires a robust infrastructure for its implementation, as well as the use of special sensors for the monitoring process. On the other hand, the work presented in [13] is characterized by the inclusion, in its telehealth process, a monitoring system oriented towards genetic counseling, mental health, and postpartum care.

Based on the review of studies presented by Josephus F. van den Heuvel [37] and in this work (see Table 1), our system is characterized differently from other proposals by the fact that it carries out remote monitoring and control of hypertensive, diabetic, and high-risk pregnant women, through specialized services, such as medical consultations, clinical history management, and the reception of medical alerts, which are generated from the data registered by the patient. In addition, our system allows for the creation of medical recommendations to the patient, and it reports alarm symptoms, which include hypertension and diabetes, among others, as well as performs infant monitoring. On the other hand, and according to the study presented in [37], it is noted that there is limited research work focused on the assessment of the usability of telemonitoring systems oriented toward high-risk pregnant women through laboratory study. Finally, also in the works presented in [37,38,39], it has been identified that there is a need to educate and raise awareness among pregnant women on various issues related to maternal–fetal risks associated with hypertension, type 2 diabetes mellitus, gestational diabetes, maternal infection, fetal malformations, among others. Particularly in the works proposed in [38,39], results have been obtained that show the benefits of educating and raising awareness among women on issues related to maternal–fetal risks associated with gestational diabetes mellitus and the application of prenatal tests for the detection of chromosomal abnormalities. On the other hand, according to the work proposed in [40], another important aspect of preventive health care for pregnant women and their newborns is the care of women’s oral health. Poor oral hygiene during pregnancy leads to the premature birth of low-birth-weight infants. Therefore, in our system, we will develop a module related to the education and awareness of women on various topics such as fetal development, pregnancy complications, healthy lifestyle during pregnancy, generic and specific guidance/advice during pregnancy, and lactation. We will also include a module aimed at monitoring and controlling the oral health of pregnant women.

## 5. Conclusions and Future Work 

In this paper, the analysis, design, development, and usability assessment of a telemonitoring system focused on the overseeing and control of hypertensive, diabetic, and high-risk pregnant women were introduced. The proposed system consists of two mobile applications, one aimed at the medical part and the other focused on the patient. Our system was validated through a usability assessment based on a laboratory study with fourteen users: seven doctors and seven patients. Based on the obtained results, patients and doctors held favorable opinions of the telemonitoring system proposed in this study. From the perspective of doctors, the main advantages of the system are the monitoring and control of high-risk pregnant women through the modules of medical consultations, clinical history management, consultation of medical alerts generated by the patient, and generation of medical recommendations to the patient. On the other hand, from the perspective of patients, the main advantages of the system are access to the information regarding their pregnancy treatment through the clinical history consultation, and to record alarm symptoms, record hypertension symptom data, record diabetes symptom data, record clinical analyses/medical studies, view recommendations provided by the specialist doctor, and the generation of alerts that are sent to the doctor, to detect risk situations to the patient’s and her infant’s health. Finally, our telemonitoring system considers and satisfies the requirements and suitable parameters that should be monitored in hypertensive, diabetic, and high-risk pregnant women according to the specialized literature. Therefore, our system can be considered a specialized software tool that could contribute to complementing the remote monitoring and control of hypertensive, diabetic, and high-risk pregnant women in healthcare institutions. On the other hand, we consider that a limitation or disadvantage of our system is that it requires an internet connection to be accessed by users. For future work and to implement our system in a public or private health institution, we consider performing usability tests with patients and physicians during the course of a year.

## Figures and Tables

**Table 1:** Comparison of telemonitoring systems.

Related Work	PA	DA	CH	MR	AG	MM	FM	DV
**Market Applications**								
[9]	✓	x	x	x	x	✓	x	✓
[10]	✓	x	x	x	x	✓	x	✓
[11]	✓	x	x	x	x	✓	x	✓
[12]	✓	x	x	x	x	✓	x	✓
**Literature Approaches**								
[13]	x	✓	✓	✓	x	✓	✓	✓
[14]	✓	✓	x	✓	x	✓	✓	x
[15]	x	✓	x	x	✓	✓	x	✓
[16]	✓	✓	✓	✓	x	✓	x	✓
[17]	x	✓	x	x	✓	✓	✓	✓
[18]	✓	✓	x	✓	x	x	✓	x
[19]	✓	✓	x	✓	✓	✓	✓	x
[20]	x	✓	x	✓	✓	x	✓	x
[21]	✓	✓	x	x	✓	✓	✓	x
[22]	x	✓	x	x	x	✓	✓	x
[23]	✓	x	x	x	✓	✓	x	x
[24]	✓	✓	✓	✓	x	x	x	✓
[25]	✓	✓	x	✓	x	x	x	x
Our work	✓	✓	✓	✓	✓	✓	✓	✓

## Data Availability

Data sharing is not applicable to this article.

## Appendix A

**Table A1:** Identification and classification of risk situation alerts.

	Alert Type						
Alert Symptom Data	Red					Yellow	
General Data							
Blood pressure (systolic/ diastolic mm Hg)	Data outside the range of 110–130/75–85				Patient reports 2 or more symptoms		
Temperature (°C)		≥38					
Heart rate (bpm)			Data outside the range of 100–60				
Glucose level (mg/dL)				(a) Preprandial Blood Glucose less than 70 or greater than 120; (b) Postprandial Blood Glucose greater than 180			
Fever							
Fever with chills	X						
Fever with sweating		X					
Breathing							
Breathing with difficulty	X						

Breathing with sound		X					

Restless breathing			Patient reports any symptoms and intensity < 6	Patient reports ≥ 4 symptoms			Patient reports between 2 and 3 symptoms
Slow breathing							
Swelling Data						Patient reports one or 2 symptoms	
Legs	Patient reports more than 2 symptoms						
Arms							
Face							
Hands							
Headache							
Intensity	≥6						
Buzzing							
with lights							
Vomit							
Dizziness							
Vaginal Fluid							
Color							
Odor							
Flow stains clothes						X	
Discomfort during Urination							
Pain	X						
Burning		X					
Bloody urine			X				
Belly Pain							
Pain with hard belly	X						
Pain with bleeding		X					
Intensity			>6	Patient reports 3 or more symptoms of pain and intensity < 6			Patient reports 0; 1 or 2 symptoms of pain and intensity ≤ 6
Localized pain							
Generalized pain							
Colic pain							
Constant pain							
Accompanied with headache							
Accompanied with waist pain							
Bleeding							
Intensity	>0						
frequency		X					
Pain in the lower belly			X				
With hip paint				X			
With waist pain				X			
Infant Movements							
Loss of fluid from the vagina	X						
Decreased movement		X					
Diabetes Symptoms							
Increased urine frequency	Patient Reports four or more symptoms					Patient reports equal or less than three symptoms	
Increased water intake							
Fatigue							
Increased sweating							
Increased nausea							
Vomit							
Experience muscle stiffness							
Feeling lightheaded experience							
Patient exhibits fruitful breath							
Patient experiences fainting sensation							
Fainting							
